# Seasonal and Long-Term Changes in Relative Abundance of Bull Sharks from a Tourist Shark Feeding Site in Fiji

**DOI:** 10.1371/journal.pone.0016597

**Published:** 2011-01-27

**Authors:** Juerg M. Brunnschweiler, Harald Baensch

**Affiliations:** 1 ETH Zurich, Zurich, Switzerland; 2 Department of Biology, University of Nebraska at Kearney, Kearney, Nebraska, United States of America; Smithsonian's National Zoological Park, United States of America

## Abstract

Shark tourism has become increasingly popular, but remains controversial because of major concerns originating from the need of tour operators to use bait or chum to reliably attract sharks. We used direct underwater sampling to document changes in bull shark *Carcharhinus leucas* relative abundance at the Shark Reef Marine Reserve, a shark feeding site in Fiji, and the reproductive cycle of the species in Fijian waters. Between 2003 and 2009, the total number of *C. leucas* counted on each day ranged from 0 to 40. Whereas the number of *C. leucas* counted at the feeding site increased over the years, shark numbers decreased over the course of a calendar year with fewest animals counted in November. Externally visible reproductive status information indicates that the species' seasonal departure from the feeding site may be related to reproductive activity.

## Introduction

Sharks and rays are increasingly popular tourist attractions, leading to growth in the popularity of marine wildlife watching as a tourism activity [1], [2]. Shark tourism contributes millions of dollars annually to local and regional economies [3]–[5], but remains controversial because of major concerns originating from the need of tour operators to use bait or chum to reliably attract certain species to specific sites [6], [7]. Despite the establishment of many shark tourism sites in recent years, baseline data on seasonal and long-term trends in shark abundance are still largely missing from such diving venues. Observational studies at shark tourism sites are important because they can provide fishery-independent scientific information on changes in shark populations, and help monitor the impact of shark attracting operations [8]–[10].

This study evaluates multi-year underwater visual, photographic and video data of bull sharks *C. leucas* from a shark feeding site in a marine protected area in Fiji. Specifically, we observe and count *C. leucas* at the feeding site in the Shark Reef Marine Reserve and address the questions: 1) What are the seasonal and long-term changes in relative abundance? 2) Based on individual identifications, how many *C. leucas* are using this feeding station? 3) What is the sex-ratio? and 4) How does the reproductive status vary seasonally? Although we do not attempt to assess the impact of baiting on *C. leucas* in this study, we provide baseline data on the long-term trend in relative abundance and seasonal cycle of the species. Overall, our results help elucidate whether the number of *C. leucas* visiting the site changed over the years, and give insight into the reproductive cycle of the species in Fijian waters.

## Materials and Methods

### Study Area and Dive Protocol

The Shark Reef Marine Reserve is a no-take zone on the southern coast of Viti Levu, Fiji, and an ecotourism project designed to protect a small reef patch and its fauna while preserving the livelihood of local communities [3]. A local dive operator began dumping fish scraps on the reef to attract sharks in 1999. Villagers who used to fish the reef, and representatives from the local dive operator, report that sightings of sharks were infrequent before feeding began. Since 2003, a single dive operator has conducted regular shark dives that include hand-feeding of up to eight different species of sharks, the numerically dominant species being *C. leucas* [11]. Two-tank dives following a specific dive and feeding protocol have taken place 3–4 times per week between 0900 and 1300 hrs. Briefly, the dive procedure starts with a first dive to 30 m where, in order to attract the sharks, a staff diver disperses small fish scraps out of a bin in front of the guests lined up behind a wall made out of dead corals. After 17 min, the divers ascend up the reef slope from 30 m to 10 m where the feeder hand-feeds grey reef *Carcharhinus amblyrhynchos* and whitetip reef sharks *Triaenodon obesus* with fish scraps and fillets. After a one hour surface interval a second dive is conducted at the same site at 16 m. Here, the feeder hand-feeds *C. leucas* and occasionally, if present, sicklefin lemon *Negaprion acutidens*, silvertip *Carcharhinus albimarginatus* and tiger sharks *Galeocerdo cuvier* with whole fish heads (mainly *Thunnus* spp. and oilfish *Ruvettus pretiosus*).

### Data Collection and Analysis

Data were collected between 2003 and 2010 using direct observation sampling methods [12]. A trained observer accompanied the tourist dives to collect data on all shark species present. Photographs and video footage were taken whenever possible to facilitate individual identification using natural marks and pigmentation [13]–[15]. For this study, the following data were considered: 1) number of *C. leucas* observed between 2003 and 2009 (recorded on 882 days; mean ± SD = 126±43.3 days per year; note that on each day, two dives of ∼40 min each separated by a one hour surface interval were conducted (see previous paragraph), and only the dive with the higher number of *C. leucas* counted was included in the analysis), 2) number of male and female *C. leucas*, determined from the presence or absence of claspers, between 2003 and 2008 (855 dives; 142.5±109.3 dives per year), 3) number of positively identified *C. leucas* between 2003 and 2009, and 4) externally visible reproductive status information in *C. leucas* between 2003 and 2010, judged from relative clasper length, mating scars and signs of pregnancy [16].

Regression analysis was used to evaluate seasonal and long-term trends in *C. leucas* relative abundance at the feeding site. Mean monthly counts were calculated and analysed by using ordinary least squares regression. To ensure independence of error terms we tested for autocorrelation in the residuals from all regression models using the Durbin-Watson statistic [17].

## Results

### Seasonal and Long-Term Changes in Relative Abundance

**Figure 1:**
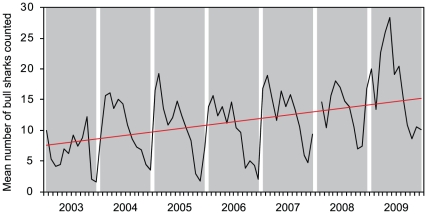
Long-term trend in relative abundance of *C. leucas* at the Shark Reef Marine Reserve, Fiji between 2003 and 2009. Regression analysis was used to evaluate a long-term trend in *C. leucas* counts at the feeding site. No data are available for January 2008. There was a long-term increase in *C. leucas* numbers (*y* = 0.0965*x*−111.71, R^2^ = 0.18, p<0.001).

**Figure 2:**
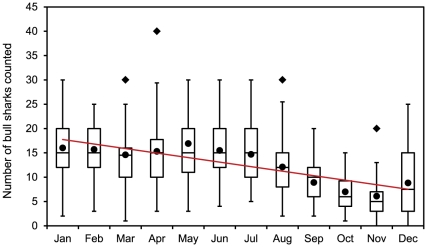
Seasonal trend in relative abundance of *C. leucas* at the Shark Reef Marine Reserve, Fiji between 2003 and 2009. Box plots show the median (line within the boxes), mean (full circles) and interquartile ranges IQR (boxes). The ends of the whisker are set at 1.5×IQR above the third quartile and 1.5×IQR below the first quartile. If the minimum or maximum values are outside this range, then they are shown as outliers (full diamonds). Regression analysis was used to evaluate a seasonal trend in *C. leucas* counts at the feeding site. There was a decrease in *C. leucas* numbers over the course of a calendar year with fewest sharks counted in November (*y* = −0.9301*x*+18.679, R^2^ = 0.7463, p<0.001).

The total number of *C. leucas* counted on each day ranged from 0 to 40 (Fig. S1) and both a long-term trend in relative abundance and seasonal cycle were observed. There was a long-term increase in *C. leucas* numbers at the feeding site (Fig. 1; *y* = 0.0965*x*−111.71, R^2^ = 0.18, p<0.001). The number of *C. leucas* counted at the Shark Reef Marine Reserve decreased over the course of a calendar year (Fig. 2; *y* = −0.9301*x*+18.679, R^2^ = 0.7463, p<0.001) with fewest sharks counted in November (mean ± SD = 6.1±4.2 *C. leucas*). Lower numbers of *C. leucas* started to be seen in August, and numbers started to increase again in December (Fig. 2). Whereas this overall pattern was observed in all years, no statistically significant decrease in *C. leucas* numbers at the feeding site was observed in the years 2003 and 2008 (Fig. S2). Days with no *C. leucas* present at the feeding site only occurred in November and December in the years 2003 (n = 1), 2005 (n = 2) and 2006 (n = 1) (Fig. S2).

### Individuals and Sex-Ratio

**Figure 3:**
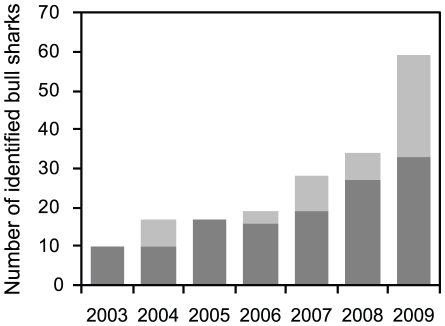
Number of individually identified *C. leucas* from 2003 to 2009. Dark grey bars denote the number of identified sharks at the beginning of the respective year; light grey bars denote the number of individuals added to the list during the respective year.

A total of 62 individual *C. leucas* were visually identified based on marks and pigmentation between 2003 and 2009 (Table S1, Fig. S3). The biggest increase in number of identified *C. leucas* compared to the previous years occurred in 2009 when 26 new individuals were added to the list of identified sharks (Fig. 3). With the exception of two individuals (“Amsterdam” and “Bite”), all animals were seen in multiple years (Table S1). One male *C. leucas* (“Jaws”) was first observed in 2003 and after being a regular visitor to the site for two years, disappeared in 2005. The mean female:male sex-ratio of positively identified *C. leucas* was 3.4, whereas the overall female:male sex-ratio was 3.6.

### Bull Shark Reproductive Status Information

*Carcharhinus leucas* encountered at the Shark Reef Marine Reserve were predominantly large animals estimated to range from >1.8 to >3 m. All male *C. leucas* observed had claspers that were elongated and extended beyond the pelvic fins (Fig. S3G and R). Females with mating scars and wounds were observed from the end of December into February. Only rarely did a male appear with a bite-mark or wound (supporting video S1). Pregnancy in females, indicated by the streamlined shape typical of the non-pregnant female becoming more rounded (Fig. S3H and I) became apparent in July and progressed until the end of the year (supporting videos S2 and S3). Individually identifiable females were observed pregnant in either odd or even years. Such females were recorded as non-pregnant when observed again at the feeding site after they left the site pregnant between October and December in the previous year.

## Discussion

Overall, *C. leucas* relative abundance at the Shark Reef Marine Reserve increased since regular feeding began in 2003 as evidenced from both the daily counts, as well as the number of individually identified sharks. A similar long-term change in relative abundance was documented at another shark watching site in the Pacific Ocean for different shark species [10]. These data show that, despite means of attracting the animals remaining constant over the years, numbers do not necessarily increase to the maximum immediately after attracting or feeding operations start, but rather continuously increase over time. Disproportionately high increases can even be observed after years of operation. Future monitoring of the shark feeding operation in the Shark Reef Marine Reserve will have to show if *C. leucas* numbers continue to increase or whether they start to level off. Any change in *C. leucas* abundance at the site will likely have direct and indirect effects on other species inhabiting or visiting Shark Reef [11]. Changes in shark abundance might, for example, affect abundance, encounter rates and/or the behaviour of other species through competitive exclusion or behaviourally mediated indirect interactions [18], [19]. Such information is crucial to obtain in order to assess the impact of shark feeding on reef ecosystems.

Abundance data of mobile fish collected using underwater visual census techniques are prone to bias [20]–[22]. For example, stationary-point-counts are often imprecise because of varying environmental conditions both during and between dives (e.g. visibility) and/or individuals might be counted several times during the same dive if they cannot be individually identified based on external markings. This becomes especially relevant when the number of individuals present increases. For example, frequencies of shark numbers recorded were shown to show signs of rounding bias [10]. Similar to this, we found a tendency for even numbers to be reported more frequently than odd numbers for counts of >5 *C. leucas*, and for counts >12 *C. leucas* there was a tendency for numbers in multiples of five to be reported (Fig. S1). Together with information on the number of positively identified individuals over the course of the study, we, however, feel confident to have adequately captured the trends and changes in *C. leucas* relative abundance at the Shark Reef Marine Reserve.

With very few exceptions, individual *C. leucas* in this study were regularly encountered at the feeding site after they were positively identified, and new individuals were regularly documented. Although large juvenile *C. leucas* were occasionally seen, the majority were large mature fish. Given the mounting evidence that many coastal shark species, including *C. leucas*, show large population declines up to functional elimination, and that at least in some cases few mature individuals seem to be left [23,24; but see also 25], our finding that the number of large *C. leucas* increased at the feeding site is encouraging. It further raises the question of where *C. leucas* attracted to the Shark Reef Marine Reserve are from. Previous research has shown that large-scale movements tend to be comparatively limited in *C. leucas* and that the species shows some fidelity to specific coastal areas [26]–[29], making the recruitment of large individuals to the Fijian feeding site from other countries in the South Pacific unlikely. Our results rather suggest that each year, more *C. leucas* from Fijian waters have come upon the feeding site and showed a certain degree of fidelity to it in subsequent years. Future studies using telemetry techniques as well as genetic analyses may confirm this conclusion, and elucidate the temporal and spatial distribution of *C. leucas* in Fijian waters including intra- and interpopulation linkages.

Whereas overall counts of *C. leucas* encountered at the Shark Reef Marine Reserve increased, the seasonal pattern of greater *C. leucas* counts in the first half of a calendar year and fewer animals in the second half, with lowest numbers counted between October and December, did not change over the course of the study. Seasonal cycles of shark abundance are also well known from other sites where sharks are attracted for tourism purposes and have been suggested to relate, at least in some species, to breeding migrations [e.g. 10], [30], [31]. The results presented in this study indicate that this hypothesis also holds for *C. leucas* in Fiji: 1) the majority of male and female *C. leucas* observed at the Shark Reef Marine Reserve were animals estimated to be well over 2 m and therefore sexually mature [32], 2) females with mating scars and wounds were only observed starting at the end of December until February, and 3) positively identified female *C. leucas* that were pregnant returned non-pregnant after being absent from the feeding site for several weeks at the end of a calendar year. Based on these observations, we conclude that the species' seasonal departure from the feeding site is related to reproductive activity and propose the following reproductive cycle for *C. leucas* in Fijian waters: mating occurs at the beginning of the calendar year; parturition at the end of a calendar year; and females mate again one year after parturition, thus completing a biennial reproductive cycle similar to other carcharhinid sharks [33]. Such a seasonal cycle would be similar to the species' reproductive cycle in the northern hemisphere where gravid adult female *C. leucas* enter nursery grounds on the east coast of Florida in late spring where parturition occurs in the summer months [34].

In Fiji, it remains unknown where mating and nursery areas of *C. leucas* encountered at the Shark Reef Marine Reserve are located. Copulation was never directly observed at the feeding site, but the quick healing of mating wounds recorded in this study and known from other shark species [15], [16], [35] suggests that mating takes place in the vicinity of the Shark Reef Marine Reserve. Additionally, several major river systems that offer suitable habitat for juvenile *C. leucas* are in close proximity to Shark Reef [36]. This indicates that relatively small areas can be effective for the protection of coastal shark species, and small-scale local conservation efforts such as the Shark Reef Marine Reserve and the Shark Corridor on the southern coast of Viti Levu, Fiji, in which shark fishing is prohibited [3], may be effective initiatives for *C. leucas* conservation.

The public debate over baiting sharks for marine tourism is largely based on inference, opinion and anecdote, primarily due to a lack of baseline data on things such as seasonal cycles and long-term trends in abundance of sharks associated with such activities. Although we did not attempt to assess the impact of baiting on *C. leucas* in this study, we provide baseline data on the long-term trend in abundance and seasonal cycle of the species at a feeding site in Fiji. Our data show that shark feeding and attracting operations can be used to collect relative abundance data that could serve as a crude monitoring instrument for conservation purposes.

## Supporting Information

Figure S1 Frequency histogram of *C. leucas* counts at the Shark Reef Marine Reserve, Fiji between 2003 and 2009.
(PDF)

Figure S2 Seasonal trends in relative abundance of *C. leucas* at the Shark Reef Marine Reserve, Fiji for the years 2003 to 2009. Box plots show the median (line within the boxes), mean (full circles) and interquartile ranges IQR (boxes). The ends of the whisker are set at 1.5×IQR above the third quartile and 1.5×IQR below the first quartile. If the minimum or maximum values are outside this range, then they are shown as outliers (full diamonds). * = statistically significant at the 1% level; ** = statistically significant at the 5% level.
(PDF)

Figure S3 Photographs showing individual *C. leucas*. (A) “Bum”; (B) “Crook”; (C) “Hook”; (D) “Stumpy”; (E) “Granma”; (F) “Rip”; (G) “Chopper” (below) and “Trevally” (above); (H) and (I) “Hotlips” photographed in April 2009 and September 2009, respectively; note the streamlined shape in (H) and the more rounded shape indicating pregnancy in (I); (J) “Bumphead”; (K) “Chica”; (L) “Detour”; (M) “Topsail”; (N) “Lee”; (O) “Junior”; (P) “Nani”; (Q) “Shorty”; (R) “Trailer”. Note the elongated claspers that extend beyond the pelvic fins in males (G) and (R) and the rounded shape indicating pregnancy in females (I) and (K). Refer to Table S1 for description of natural marks of individuals. All photographs are copyright to Lill Haugen.
(PDF)

Video S1 A male *C. leucas* (“Bite”; note claspers) with a fresh bite mark on its right side just behind the corner of the mouth documented at the Shark Reef Marine Reserve, Fiji. Refer to Table S1 for description of natural marks of individuals.
(M4V)

Video S2 A female *C. leucas* (“Granma”) documented in January 2004 at the Shark Reef Marine Reserve, Fiji. Refer to Table S1 for description of natural marks of individuals.
(M4V)

Video S3 The same (see video S2) female *C. leucas* individual documented in November 2004 at the Shark Reef Marine Reserve, Fiji. Note the rounded shape indicating pregnancy compared to the more streamlined shape in video S2 typical of the non-pregnant female.
(M4V)

Table S1 Description of 62 *C. leucas* visually identified between 2003 and 2009 (terminology of technical terms follows £).
(PDF)
