# Supplementary figures and images for: Seasonal and Long-Term Changes in Relative Abundance of Bull Sharks from a Tourist Shark Feeding Site in Fiji
Source: PLoS One. 2011 Jan 27;6(1):e16597. doi: 10.1371/journal.pone.0016597 (PMC3029404; doi:10.1371/journal.pone.0016597)

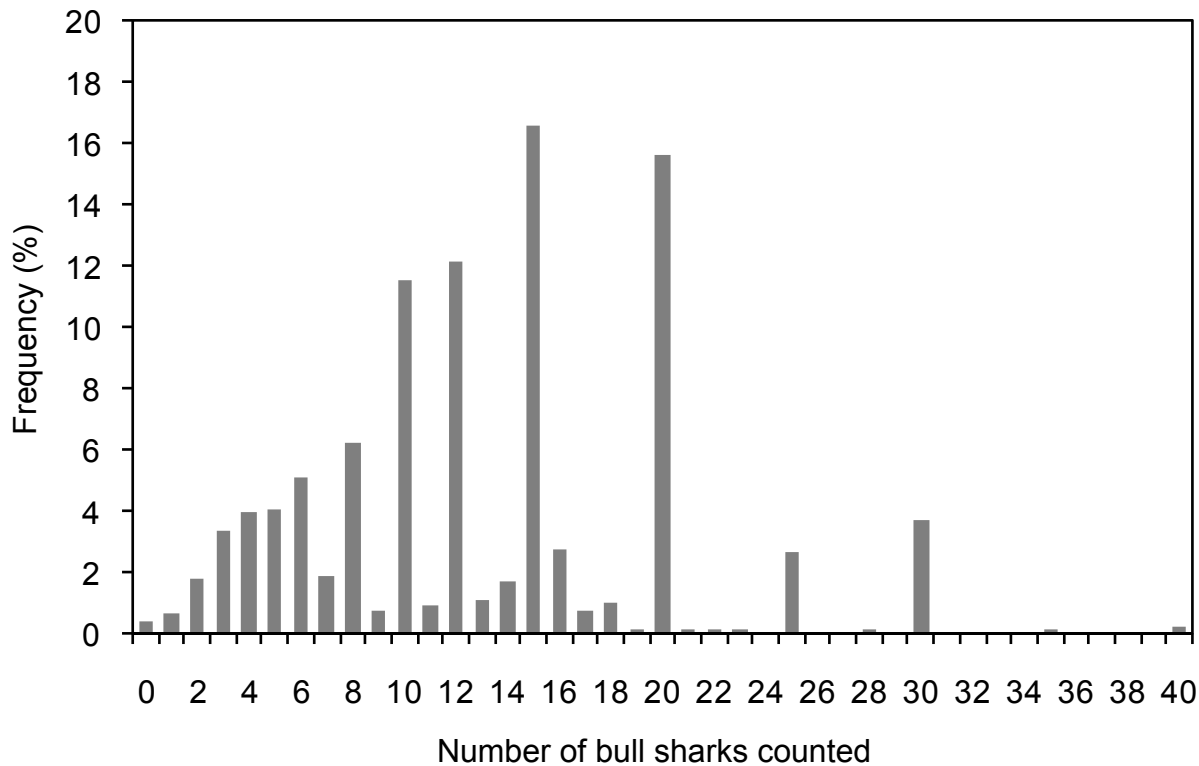

Supplement: Figure S1 — Frequency histogram of C. leucas counts at the Shark Reef Marine Reserve, Fiji between 2003 and 2009. (PDF) [file pone.0016597.s001.pdf]

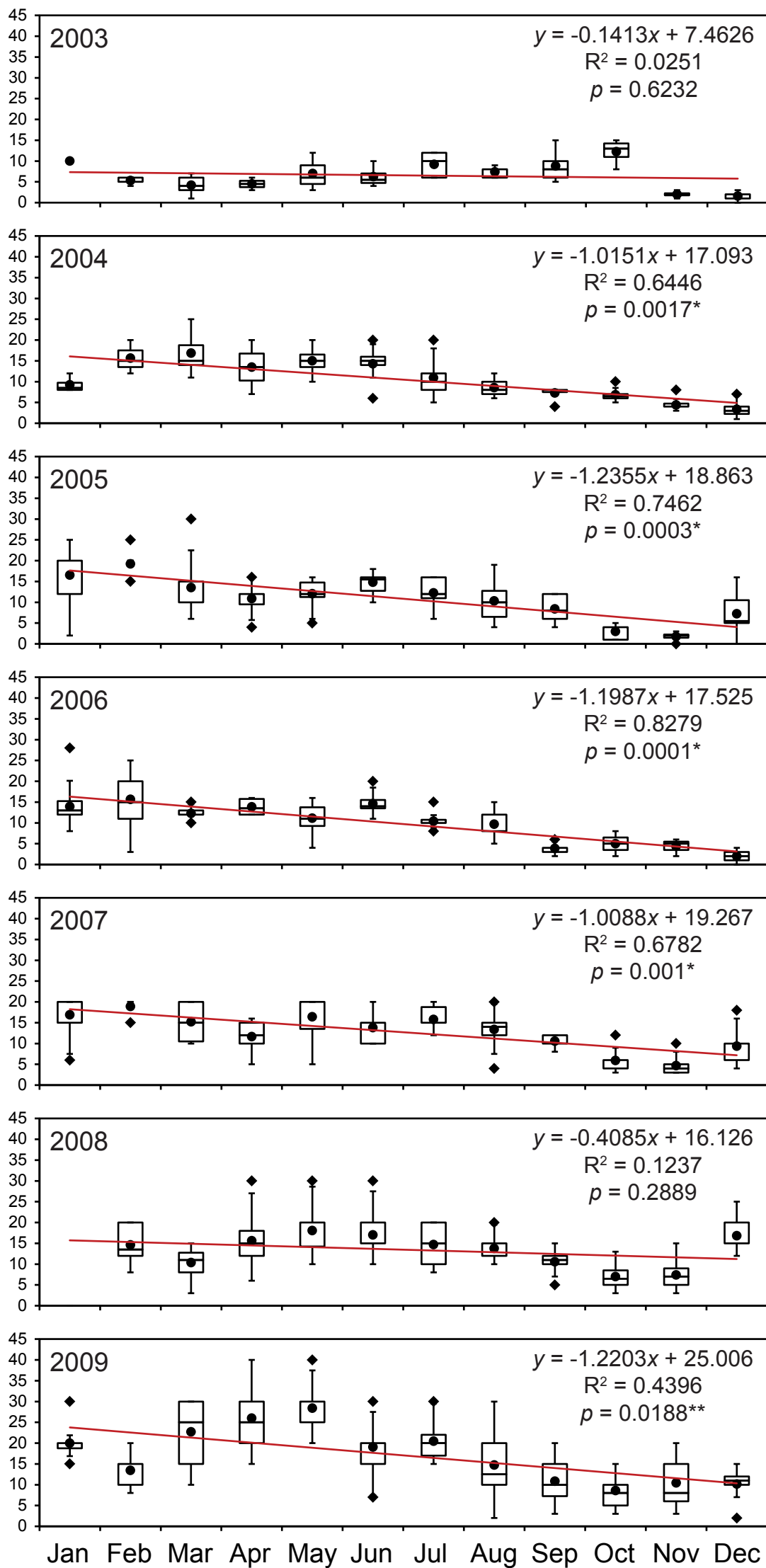

Supplement: Figure S2 — Seasonal trends in relative abundance of C. leucas at the Shark Reef Marine Reserve, Fiji for the years 2003 to 2009. Box plots show the median (line within the boxes), mean (full circles) and interquartile ranges IQR (boxes). The ends of the whisker are set at 1.5×IQR above the third quartile and 1.5×IQR below the first quartile. If the minimum or maximum values are outside this range, then they are shown as outliers (full diamonds). * = statistically significant at the 1% level; ** = statistically significant at the 5% level. (PDF) [file pone.0016597.s002.pdf]

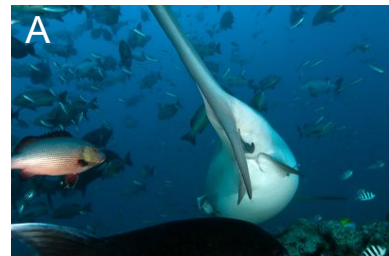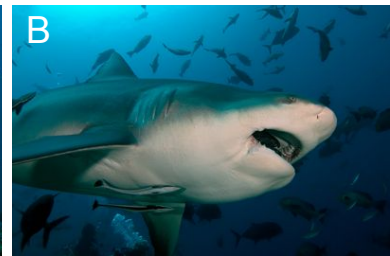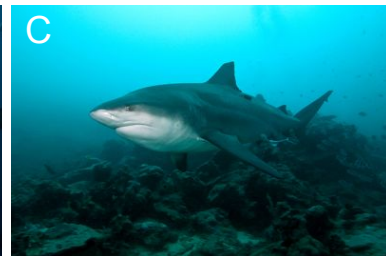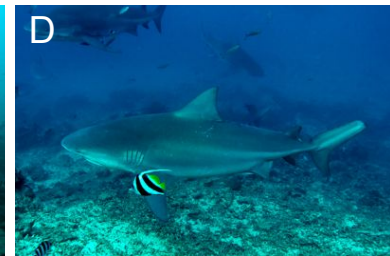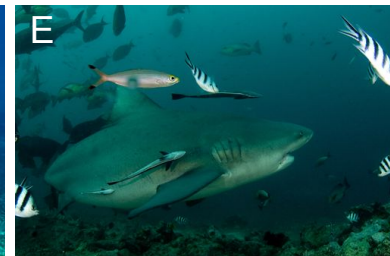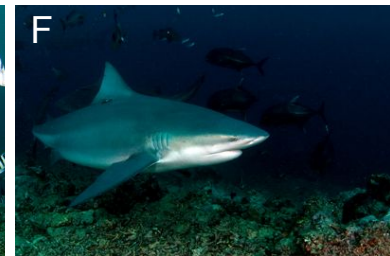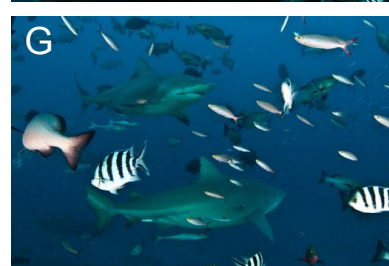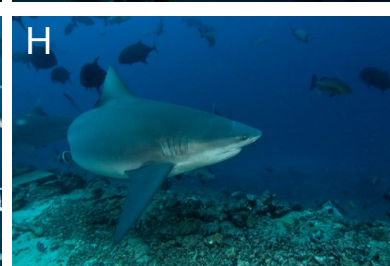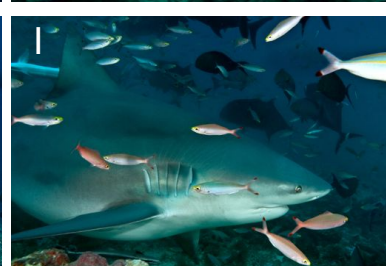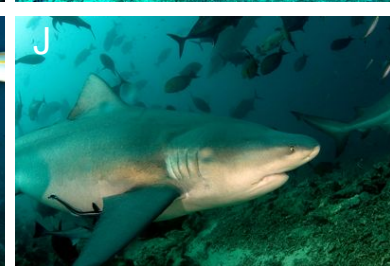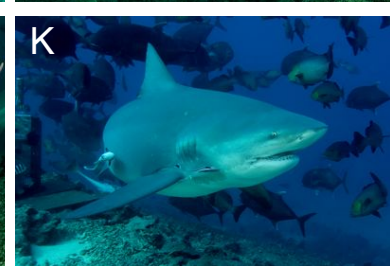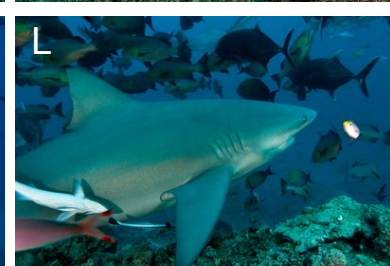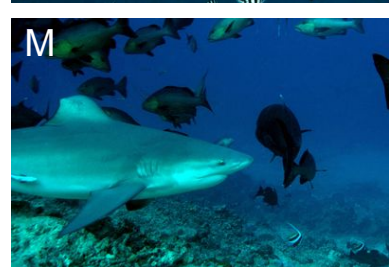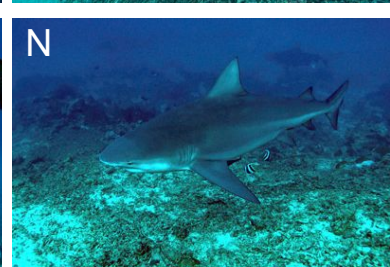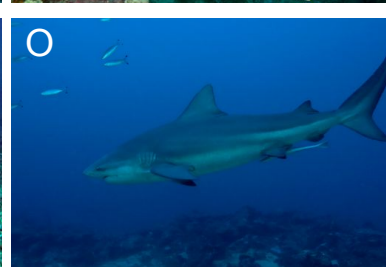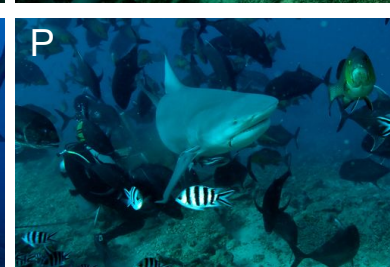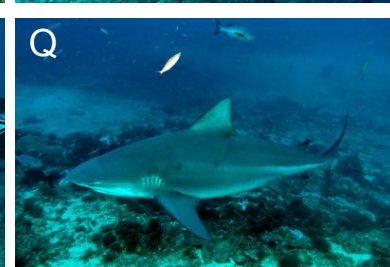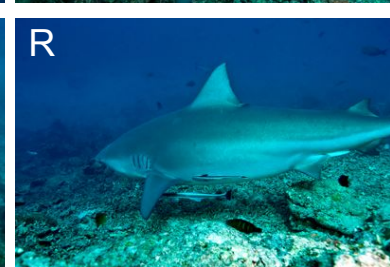

Supplement: Figure S3 — Photographs showing individual C. leucas. (A) “Bum”; (B) “Crook”; (C) “Hook”; (D) “Stumpy”; (E) “Granma”; (F) “Rip”; (G) “Chopper” (below) and “Trevally” (above); (H) and (I) “Hotlips” photographed in April 2009 and September 2009, respectively; note the streamlined shape in (H) and the more rounded shape indicating pregnancy in (I); (J) “Bumphead”; (K) “Chica”; (L) “Detour”; (M) “Topsail”; (N) “Lee”; (O) “Junior”; (P) “Nani”; (Q) “Shorty”; (R) “Trailer”. Note the elongated claspers that extend beyond the pelvic fins in males (G) and (R) and the rounded shape indicating pregnancy in females (I) and (K). Refer to Table S1 for description of natural marks of individuals. All photographs are copyright to Lill Haugen. (PDF) [file pone.0016597.s003.pdf]
