# Supplementary material for: Seasonal and Long-Term Changes in Relative Abundance of Bull Sharks from a Tourist Shark Feeding Site in Fiji
Source: PLoS One. 2011 Jan 27;6(1):e16597. doi: 10.1371/journal.pone.0016597 (PMC3029404; doi:10.1371/journal.pone.0016597)
Supplement: Table S1 — Description of 62 C. leucas visually identified between 2003 and 2009 (terminology of technical terms follows £). (PDF) [file pone.0016597.s007.pdf]

Table S1. Description of 62 *C. leucas* visually identified between 2003 and 2009 (terminology of technical terms follows £).

Name	Sex	Description	First seen	Last seen
Annie	F	Tip of anal fin cut horizontally	2003	2010
Bum*	F	Anal fin curled to the right	2003	2010
Crook*	F	Both corners of mouth crooked	2003	2010
Hook*	F	Apex of first dorsal fin cut; cut slanting forwards	2003	2010
Kinky	F	Kink in anterior margin of first dorsal fin	2003	2010
Stumpy*	F	Terminal lobe of caudal fin missing	2003	2010
Jaws	M	Broken left lower jaw with tracer grown into corner of mouth	2003	2005
Long John	M	Terminal lobe of caudal fin cut with s-shaped axial bend below cut	2003	2010
Whitenose	M	White small horizontal blotch on tip of snout	2003	2010
Flop	F	Terminal lobe of caudal fin slightly bent to the left	2004	2010
Granma*$	F	Both corners of mouth crooked (right side with distinctive long vertical cut); bad right eye; very long pectoral fins; right pectoral fin with upward swing	2004	2010
Grin	F	Right corner of mouth crooked	2004	2010
Monica	F	Terminal lobe of caudal fin ridged and curled to the right	2004	2010
Rip*	F	Rip on posterior margin of first dorsal fin	2004	2010
Blackbeard	M	Large; hook in right corner of mouth	2004	2010
Chopper*	M	Dorsal lobe of caudal fin cut; cut ragged resulting in zig-zag edge	2004	2010
Hotlips*	F	White horizontal bulge on right labrial furrow	2006	2010
Second	F	Apex of second dorsal fin cut horizontally	2006	2010
Tipper	F	Tip of terminal lobe of caudal fin folded over to the right	2006	2010
Alexis	F	Second dorsal fin missing	2007	2010
Bumphead*	F	Head stunted; snout bent upwards as if shark had bumped into an obstacle	2007	2010
Chica*	F	Right upper corner of mouth with narrow bulge protruding forward	2007	2010
Detour*	F	Posterior margin of first dorsal fin curving inwards, then outwards, then back inwards (a "detour")	2007	2010
Elvira	F	Left pelvic fin cut horizontally	2007	2010
Rusty	F	Rusty colored dot on right corner of mouth; apex of first dorsal fin rounded	2007	2010
Topsail*	F	Upper half of first dorsal fin missing; horizontal bulge below cut	2007	2010
Valerie	F	Tip of left pectoral fin bent pointing downwards	2007	2010
Amsterdam	M	Highly contrasting delimitation between dark dorsal and light ventral coloration; dark blotch on snout and one dark central blotch situated ventrally in line with posterior margins of pectoral fins	2007	2007
Crease	F	Apex of first dorsal fin slightly folded to the left along a sharp crease	2008	2010
Curly	F	Apex of first dorsal fin curled/folded to the left	2008	2010
Lee*	F	Dorsal lobe of caudal fin cut vertically; cut ragged resulting in zig-zag edge	2008	2010
Line	F	Apex of first dorsal fin cut	2008	2010
Maite	F	Apex of second dorsal fin curled to the left	2008	2010

Scar	F	Vertical scar on the right side of the body below first dorsal fin; bright white eyes	2008	2010
Bite#	M	Shark bite on right side of the head above right corner of mouth	2008	2008
Big Mama	F	Very large; posterior margin of first dorsal fin cut and ragged; apex cut with cut slanting forward; right hand corner of mouth crooked	2009	2010
Blunt	F	Apex of both pectoral fins blunted	2009	2010
Brenda	F	Free rear tip and inner margin of first dorsal fin cut	2009	2010
Gill	F	Notable wide wound on first gill slit (right side)	2009	2010
Helen	F	Mole-like circular scar at corner of mouth (right side)	2009	2010
Jennifer	F	Small female with wound on lower jaw (right side)	2009	2010
Junior*	F	Small; anal fin atrophied	2009	2010
Lill	F	Small; horizontal notch in lower postventral margin of caudal fin	2009	2010
Marlen	F	Anterior margin of apex of first dorsal fin kinked and slightly buckled to the right	2009	2010
Miss America	F	Half of dorsal lobe of caudal fin missing; anal fin atrophied	2009	2010
Moana	F	Large; left corner of mouth deeply crooked; right corner of mouth mildly crooked	2009	2010
Nani*	F	Large; right corner of mouth with deep, long vertical incision; apex of right pectoral fin bent upwards at a 90 degree angle	2009	2010
Naughtylus	F	Huge; right corner of mouth slightly crooked; small hooks in each corner of mouth; posterior margin of apex of first dorsal fin slightly notched	2009	2010
Nick	F	Missing chunk on posterior margin of first dorsal fin; bad left eye	2009	2010
Noodles	F	Noodle-like thick fishing line protruding from below right side of mouth	2009	2010
Pointer	F	Apex of first dorsal fin with distinctive sickle-shaped point	2009	2010
Shorty*	F	Free rear tip of first dorsal fin cut	2009	2010
Sickle	F	Small; characteristic short and sickle-shaped first dorsal fin	2009	2010
Sierra	F	Left corner of mouth crooked	2009	2010
Tip	F	Small portion of tip of apex of first dorsal fin chipped and bent to the right; rounded first and second dorsal fins; terminal lobe of caudal fin blunted	2009	2010
Tipless	F	Terminal lobe of caudal fin missing	2009	2010
Trevally*	F	Large; keel-like protuberance on ventral lobe (right side) of caudal fin	2009	2010
Twist	F	Inner margin of first dorsal fin twisted	2009	2010
Wave	F	Characteristic bulge on lower postventral margin of caudal fin	2009	2010
Sharkman	M	Large; right corner of mouth with circular scar	2009	2010
Trailer*	M	Free rear tip of first dorsal fin twisted	2009	2010

£ Compagno LJV (2001) Sharks of the world. An annotated and illustrated catalogue of shark species known to date. Volume 2. Bullhead, mackerel and carpet sharks (Heterodontiformes, Lamniformes and Orectolobiformes). FAO Species Catalogue for Fishery Purposes. No. 1, Vol. 2. Rome: FAO. 269p.

* See Fig. S3 for photograph.

# See supporting video 1.

$ See supporting videos 2 and 3.
